# Calibrating the Test of Relational Reasoning: New Information From Oblique Bifactor Models

**DOI:** 10.3389/fpsyg.2020.02129

**Published:** 2020-09-02

**Authors:** Denis Federiakin

**Affiliations:** ^1^Center for Psychometrics and Measurement in Education, Institute of Education, National Research University Higher School of Economics, Moscow, Russia

**Keywords:** relational reasoning, the test of relational reasoning, bifactor models, oblique bifactor models, the Extended Testlet Model, the Generalized Subdimensional Model

## Abstract

Relational reasoning (RR) is believed to be an essential construct for studying higher education learning. Relational reasoning is defined as an ability to discern meaningful patterns within any stream of information. Nonetheless, studies of RR are limited by the psychometric structure of the construct. For many instances, the composite nature of RR has been described as a bifactor structure. Bifactor models limit possibilities for studying the inner structure of composite constructs by demanding orthogonality of latent dimensions. Such assumption severely limits the interpretation of the results when it is applied to psychological constructs. However, over the last 10 years, advances in the fields of Rasch measurement led to the development of the oblique bifactor models, which relax the constraints of the orthogonal bifactor models. We show that the oblique bifactor models exhibit model fit, which is superior compared to the orthogonal bifactor model. Then, we discuss their interpretation and demonstrate the advantages of these models for investigating the inner structure of the test of RR. The data are a nationally representative sample of Russian engineering students (*N* = 2,036).

## Introduction

Contemporary studies of higher education learning are unthinkable without studies of cognitive processing. Over the past 20 years, educational experiments have advanced our understanding of the intellectual and moral development of students. Moreover, they also have merged educational research with cognitive field (e.g., De Clercq et al., 2013). Researchers more and more tend to explain educational phenomena in terms of information processing and higher-order thinking skills.

Among all higher-order thinking skills, relational reasoning (RR) appears to be one of the most important. Relational reasoning is defined as an ability to discern meaningful patterns within any stream of information (Alexander and The Disciplined Reading and Learning Research Laboratory, 2012; Dumas et al., 2013). The importance of RR is well-established in the educational context; RR has been utilized as a predictive measure in a variety of studies. For example, it can predict SAT scores both for the verbal section and for the mathematics section (Alexander et al., 2016a). Relational reasoning also demonstrated high levels of predictive validity in the domain of engineering design (Dumas and Schmidt, 2015; Dumas et al., 2016) and medical education (Dumas, 2017). In general, it proved to be a significant predictor of students’ ability to produce innovations and solve problems.

As with many other conceptualizations of higher-order thinking skills, RR has been suggested as a composite construct that has many parts. However, some of the most critical manifestations of it are analogy, anomaly, antinomy, and antithesis (Alexander et al., 2016a; Dumas and Alexander, 2016). Each manifestation corresponds to a particular pattern within a set of information. Although researchers can saturate these specific forms of relations with various details of relationships within a set of information elements, these patterns are usually described (Alexander et al., 2016b) as follows:

• Similarity (identifying convergence of change patterns);
• Discrepancy (identifying dissimilarity between one element and all others or finding where the pattern breaks);
• Incompatibility (defining criteria for similarity or dissimilarity and consequently, determining how to classify the elements); and
• Polarity (identifying opposites of continuum and divergence).

However, studies of RR are limited by the psychometric structure of the construct. For many instances, the composite nature of RR has been described as a bifactor structure (Dumas and Alexander, 2016). Although bifactor modeling gained much attention in recent years, its usefulness for practitioners remains somehow restricted by its interpretation and challenges in technical applications (Bonifay et al., 2017). The main problems with it are constraints introduced in the variance–covariance matrix of latent dimensions. This severe assumption is necessary for model identification and avoiding technical difficulties. However, during a recent peak of attention to these models in psychometric literature, several extensions have been proposed to relax this limitation and provide more flexible setups for modeling bifactor structures.

**FIGURE 1:**
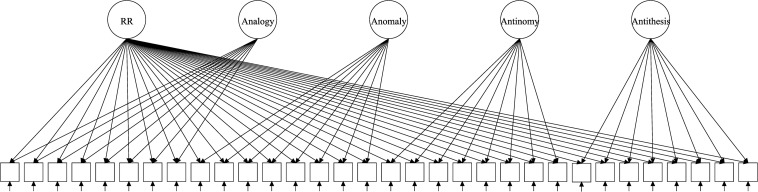
Exemplary path diagram for orthogonal bifactor model with four specific factors.

**FIGURE 2:**
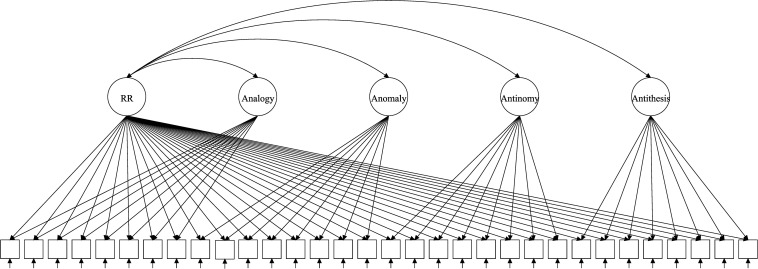
Exemplary path diagram for the ETM with four specific factors.

**FIGURE 3:**
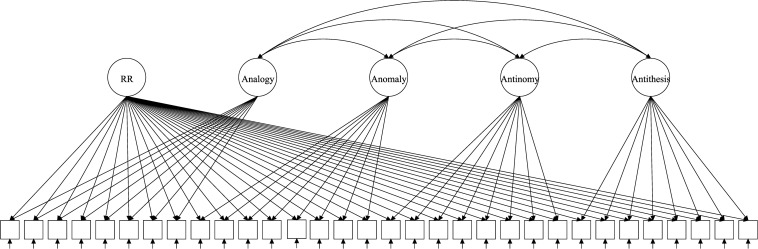
Exemplary path diagram for the GSM with four specific factors.

The test of RR (TORR) was designed (Alexander, 2012) and validated (Alexander et al., 2016a) to capture RR and its four manifestations. The TORR was calibrated within classical test theory, item response theory (IRT) and Bayesian networks (Alexander et al., 2016a; Dumas and Alexander, 2016; Grossnickle et al., 2016). Overall, the TORR has good psychometric properties and promising implementations in educational studies. The measure has 32 nonverbal items organized into four 8-item scales that represent the four forms of RR (Figures 1–3 reflect the structure of the TORR under different model assumptions). All items are scored dichotomously and have multiple-choice formats with four response options. Additionally, each TORR scale includes two relatively easy sample items designed to familiarize participants with the content of the tasks.

The authors chose the bifactor structure of the TORR, reflecting the theoretical structure of the construct. An investigation of the TORR’s dimensionality argued that a 3PL bifactor model was the best-fitting MIRT model, within which the test was calibrated (Dumas and Alexander, 2016). However, the applied model fixates the correlations of all person-specific parameters at zero, so it is impossible to study the relations between the subcomponents of RR. Therefore, some research questions on RR could not be posed despite being of interest.

This study aims to enrich the best of our understanding of RR by advancing modeling techniques used to describe the construct. To do so, we apply oblique bifactor models, which impose less strict constraints on the variance–covariance matrix. One of these models is the Extended Testlet Model, which allows specific factors to correlate with the general factor, but forces them to be orthogonal to each other (Paek et al., 2009). Another model is the Generalized Subdimensional Model (GSM) (Brandt and Duckor, 2013), which forces specific factors to be orthogonal to the general factor but allows them to correlate with each other. We discuss the differences in their interpretation and some technical application. Then, we compare the models in terms of their model fit and estimated variance–covariance matrix and review the results obtained using the nonverbal TORR (Alexander et al., 2016a). We conclude this article with a discussion of limitations and possible further research.

The discussed models have been proposed and studied within the paradigm of Rasch measurement. Therefore, all considered models belong to Rasch measurement paradigm to make comparison across them feasible. Because the TORR utilizes dichotomous scoring, we consider only dichotomous versions of the bifactor models. Additionally, all illustrative path diagrams in the description of the models follow the structure of the TORR: 32 dichotomous items divided into four subscales (eight items per subscale).

### Bifactor Models

Bifactor models have a long history in factor analysis (Holzinger and Swineford, 1937; Schmid and Leiman, 1957). Their main feature is that each item loads on the general dimension (we call it “general factor”) and a latent variable defined by a subscale to which an item belongs (we call it “specific factor”). Such structures are useful for modeling composite instruments with non-ignorable local item dependence (LID; Bradlow et al., 1999). Local item dependence implies that item responses are random once values of all latent dimensions are known. As a result of this logic, bifactor IRT model (Bayesian Testlet Model) has been proposed, which attempted to add on latent extra dimensions to make the responses random controlling for them as well as for the general factor.

Nonetheless, such models are overparametrized and cannot be estimated unless the latent dimensions are constrained to be orthogonal (Figure 1). Assumption of total orthogonality of dimensions proposes a problem because it severely restricts the interpretation of the results. Total orthogonality means that specific factors are independent of each other and the general factor. Even if the general factor still can be somehow interpreted as the target dimension of interest, it is “purified” from components defined by specific factors. However, interpretation of specific factors becomes even more complicated, because they become purified from general RR as well as other components. Further, difficulties in interpretation of such scores met with typically low estimates of their reliability, making the subscores virtually useless (Haberman, 2008).

As a result of this, reasonable setup for bifactor modeling appears to be limited to modeling of LID in educational testing. These (constrained) correlations of specific factors describe correlations of person–testlet interactions (nuisance dimensions) and therefore are not in the focus of interest (e.g., Reise et al., 2010). However, for psychological studies, this remains somewhat questionable assumption because researchers typically expect latent dimensions to correlate (Reise, 2012). A specific example of a consistent application of bifactor models in psychological studies can be an attempt to separate a complex construct from its contexts or situations in which it manifests itself. However, it makes subscores barely useful either way. In the end, as Reise et al. (2010) noted, “researchers view bifactor structures with great suspicion” because of such interpretational difficulty.

A direct example of such approach in Rasch measurement is the original Rasch Testlet Model (Wang and Wilson, 2005). For dichotomous items, Rasch Testlet Model can be represented as

$$g\,\left(\pi_{p\,i}\right)=\theta_{p}+\gamma_{p\,\left(d\right)}-\delta_{i}$$

where, π_*pi*_ is the probability that the person *p* gets item *i* correctly, *g*(.) is a function of choice (in this study, we used inverse logit function), θ_*p*_ is the ability level of the person *p* on the general factor, γ_*p(d)*_ is an auxiliary ability level of the person *p* on the testlet-specific dimension *d*, and δ_*i*_ is the generalized difficulty of the *i*th item.

As initially proposed, person parameters are assumed to follow independent normal distributions. Variance of specific factor accumulates the dependency between the items creating the *d*th testlet (LID on θ). This parameter varies across persons and remains fixed for all items in a testlet *d*; i.e., it denotes person–testlet interaction. Thus, the probability of a correct response of person *p* on item with difficulty δ_*i*_ depends on the sum of two person-specific parameters:θ_*p*_ and γ_*p(d)*_. As a result of such decomposition, there are two points to note in interpreting the model. First, even under a low level of the general factor, person *p* can perform well for some particular testlet *d* if person *p* has a relatively high factor score on the corresponding specific factor. Second, the general factor and all specific factors are assumed to be unidimensional.

For the TORR example, orthogonal bifactor model implies that a general factor of RR is abstract, independent of its manifestations (analogy, anomaly, antinomy, and antithesis) and loads items simultaneously with them. However, this assumption is questionable, taking into account the nature of the construct. For example, commonly, researchers conceptualize the search of analogies as a basis for all cognitive functions (e.g., James, 1890; Spearman, 1927; Sternberg, 1977). Regarding four studied manifestations of RR, it means that all of them can be seen as “analogical reasoning plus something extra,” where the subscales differ in additional cognitive operations. Thus, anomaly subscale can be seen as a subscale measuring skill to find what is similar among all elements except one. Antinomy can be seen as a skill to find similarities of an initial element with secondary elements. Then, the correct answer can be determined by exclusion. Antithesis can be seen as a skill to find similarities of an initial element with secondary elements while keeping in mind a rule-implied change and reversing it. So, some elements of analogical reasoning can be found everywhere. Therefore, researchers can expect some nonzero correlations between analogy subscale and all other subscales, which has been established earlier (e.g., Alexander et al., 2016a). At the same time, the orthogonal bifactor model extracts the general factor, which can be severely contaminated by analogical reasoning.

However, such logic can be applied even further, to all other subscales. For example, antinomy subscale can be seen as a search for the anomaly, when the anchor element is presented. In contrast, in anomaly subscale itself, a respondent is required to infer the similarities across elements without the anchor. Antithesis can be seen as a search for multiple anomalies simultaneously, and so on. Therefore, nonzero correlations are expected from all subscales, which is also the case for the correlated factors model without the general factor (Alexander et al., 2016a). As a result of this, the general factor in the orthogonal bifactor model describes nothing more than a commonality between subscales of the TORR. However, if the generalized ability of RR itself is more than a positive manifold between different types of cognitive operations, the orthogonal bifactor model is not the best choice to describe it.

### The Extended Rasch Testlet Model

As an attempt to overcome limitations of the original bifactor models, Paek et al. (2009) proposed the Extended Rasch Testlet Model (ETM). The key features of this model are correlations of specific factors with the general factor (Figure 2). Consequently, specific factors are purified from each other, but they share some estimated portion of variance with the general factor. Note, that correlations of latent variables can be negative, because items from all subscales define the general factor.

The ETM has the same formulation as the original Rasch Testlet Model and only differs in the assumption applied to the correlations of person-specific parameters. Constraining all covariances between the general factor and specific factors to zero will return a variance–covariance matrix for the original Rasch Testlet Model with the corresponding structure of the testlets. Therefore, the orthogonal Rasch Testlet Model is nested within the ETM. However, the ETM should recover factor scores better than the original Testlet Model because it takes into account the shared variance of person parameters.

It is possible to interpret correlations between specific factors and the general factor as relations between specific subparts of a more general construct and general ability itself controlling for other subparts of the construct. This interpretation follows from the classical interpretation of regression analysis. These correlations may be seen as partial correlations or standardized regression coefficients from a multivariate linear regression model.

For the TORR example, the ETM implies that the general factor of RR preserves correlations with the manifestations of it. Therefore, ETM allows for a tailored test of the hypothesis whether the general factor is just a positive manifold of specific factors or not (Van Der Maas et al., 2006). If the general factor of RR preserves nonzero correlations with specific factors of it, then they indeed measure specific manifestations of RR, and the general factor is not an exhaustive descriptor of the latent space of the construct. At the same time, if the correlations of subscales with the general factor become insignificantly different from zero, then the general factor of the orthogonal bifactor model describes nothing more and then a commonality between subscales, and not a specific variable with distinct psychological interpretation. Testing this hypothesis is important because pushing general factor models beyond their limits can lead to the creation of such controversial phenomena, as a general factor of personality (e.g., Revelle and Wilt, 2013).

### The Generalized Subdimensional Model

The GSM (Brandt, 2017) is also a derivative of the original Rasch Testlet Model but in the opposite direction compared to the ETM. Instead of assuming orthogonality between specific factors, it allows them to correlate (Figure 3). Nonetheless, for model identification purposes and to ensure that specific factors represent subscale-specific components of general ability within it, several additional constraints must be made (for details, see Brandt, 2008). They regard to “translation” parameters (*k*_*d*_) weighting the variances of specific factors in order to equalize them: the sum of squares of translation parameters is constrained to be equal to the number of specific factors (*D*, for details, see Brandt and Duckor, 2013). The GSM can be described (Robitzsch et al., 2020) as

$$g\,\left(\pi_{p\,i}\right)=k_{d}\,\left(\theta_{p}+\gamma_{p\,\left(d\right)}-\delta_{i}\right).$$

Note that the GSM requires skipping one of the specific factors to avoid overconstraining (Brandt, 2008). This is achieved by defining the skipped specific factor as a negative sum of all remaining specific factors. Because one of the specific dimensions is excluded from the calibration, it is necessary to recalibrate the model with alternative reparameterizations at least three times to gather the full variance–covariance matrix of the dimensions, e.g.,

(1) Excluding the last *D*th dimension to recover all covariances between all dimensions but covariances with dimension *D*,
(2) Excluding dimension *D*-1 to recover all covariances of dimension *D* but the covariance of dimension *D* with dimension *D*-1, and
(3) Excluding dimension *D*-2 to recover the covariance of dimensions *D* and *D*-1.

A direct interpretation of this model assumes that specific factors are not purified from each other, but they are allowed to correlate freely (even negatively). Therefore, this model describes how specific factors relate to each other after the general factor is extracted. Brandt and Duckor (2013) recommended interpreting the general factor as a shared variance of dimensions from a truly multidimensional construct.

Within the context of TORR, this model describes differences in commonalities between the subscales. After the general RR is extracted, this model reveals how similar or how different the used subscales are and what is the degree of shared cognitive processing that they provoke. The correlations close to zero will mean that the subscales are virtually independent controlling for the general RR, and *vice versa*. Note that these relations are not the same as with correlated factors model, where the general factor is distributed across subscales, causing possible positive correlations. GSM explicitly models “residual” correlations between subscales, which are not described by the general factor.

When comparing the ETM and the GSM, it is important to distinguish their purposes: they are meant to answer different research questions in terms of studying the internal structure of composite constructs. These two models complement each other in terms of their focus of interest. Usage of them in a directly competitive manner fits only for deciding which model orders respondents better by the general factor. Note, however, that they extract different factor structures. This happens because of differences in constraints imposed on the variance–covariance matrix. While orthogonal testlet models and the GSM describe general RR, which is independent of its manifestations, the ETM describes general RR, which is correlated to them. Moreover, the ETM and orthogonal testlet models describe specific factors that are independent of each other. In contrast, the GSM describes specific factors that share some portion of variance with each other.

Roughly all of these models are special cases of the multidimensional random coefficients multinomial logit model (MRCMLM; Adams et al., 1997). Therefore, the TAM package for R software (Robitzsch et al., 2020) can be used to calibrate these models. Although the GSM itself is not a special case of MRCMLM (Brandt, 2017), its predecessor—the Rasch model with subdimensions (Brandt, 2008)—is. Therefore, all discussed models can be calibrated with TAM package, using the same algorithms for likelihood estimation. The parameters were estimated with the quasi–Monte-Carlo algorithm implemented in the TAM package, which proved to be efficient in the presence of high-dimensional latent ability space (Wu et al., 2007). To estimate reliability, we used expected *a posteriori* (EAP) estimates of factor scores (Bock and Mislevy, 1982) because of their flexibility in complex multidimensional setup. Moreover, EAP uses distributional information from the variance–covariance matrix to increase the precision of the estimates.

To demonstrate the advantages of oblique bifactor models in terms of global model fit, we analyzed absolute and relative model fit indices. To estimate the absolute global fit, we used root mean square error of approximation (RMSEA; Steiger, 1990) and standardized root mean square residual (SRMSR; Hu and Bentler, 1999) according to the recommendations given by Shi et al. (2020). Root mean square residual can be interpreted as an unstandardized measure of the distance between the data-generating model and the hypothesized model. Standardized root mean square residual possesses a straightforward interpretation: it is just on average of correlation residuals. As a result of this, models with lower values of these indices are preferable. We also used comparative fit index (CFI; Bentler, 1990) as an additional measure of incremental model fit. In contrast to RMSEA, CFI is commonly interpreted as a measure of the distance between the hypothesized model and the baseline model, where all the variables are uncorrelated. Therefore, models with higher CFI values are preferable. Note, however, that despite conventional “rules of thumb” derived in factor analytical approach, there are no strict cutoff criteria for IRT models (e.g., Maydeu-Olivares, 2013; Savalei, 2018; Xia and Yang, 2019). Consequently, we cannot definitively conclude that some or all models fit or do not fit the data. Additionally, we compared the relative fit of the models with the Akaike Information Criterion (AIC; Akaike, 1974) and Bayesian Information Criterion (BIC; Schwarz, 1978). These indices allow for comparison of model fit across nonnested models, introducing a penalty for extra parameters (AIC) with respect to sample size (BIC). Lower values of these indices imply a better global model-data fit accounting for model complexity.

## Data

The data used for this study is a part of a larger project, called the Super Test Project, led by researchers at Stanford University in collaboration with ETS and researchers from various countries including China and Russia. The overall purpose of this project is to examine learning outcomes and institutional- and individual-level factors related to them for electrical engineering and computer science students across multiple countries. To this end, the research team also collected a wealth of contextual survey data from students, faculty, and administrators.

As a part of the Super Test Project, the TORR was administered to Russian electrical engineering and computer science students. We randomly included 34 Russian universities in a nationally representative sample of engineering students. The testing was conducted in November–December 2016 among students graduating in 2017 (when they were in the middle of their fourth year of studying) and in April 2017 among students graduating in 2019 (when they were at the end of their second year). The testing was conducted in a computer-based format. Students had 60 min to complete the TORR. The data cleaning procedure included the deletion of all response profiles with 50% or less of the responses on any subscale. Consequently, 76 profiles were deleted from the database (approximately 3.6%). We compared correlations between subscales in raw scores before and after deletion of the profiles, to prove that the deleted responses did not bias the subsequent analyses. The change in correlations was less than 0.001. The final sample size is 2,036 students.

## Results

The results of the global model fit analysis are reported in Table 1 (note that the deviance statistic in the GSM is averaged over its four reparameterizations). As Table 1 suggests, both the ETM and the GSM fit data better, indicating that oblique bifactor models provide a better description of RR than orthogonal bifactor model. In other words, correlations of latent dimensions should not be ignored while studying RR.

**TABLE 1:** Results of the model comparison.

**Statistics**	**Models**		
	**Testlet model**	**ETM**	**GSM**
χ^2^ statistics for the baseline model		10, 980.342	
Degrees of freedom for χ^2^ statistics		496	
Sample size		2, 036	
Number of free parameters	37	41	42
Degrees of freedom for χ^2^ statistics	491	487	486
χ^2^ statistics	3, 290.570	2, 315.269	2, 299.300
RMSEA	0.053	0.043	0.043
CFI	0.733	0.826	0.827
SRMSR	0.058	0.051	0.050
Deviance	82, 009.13	81,677.74*	81, 661.43
AIC	82, 083.13	81, 759.74	81, 745.43
BIC	82, 291.52	81, 990.65	81, 981.97

*Likelihood ratio test reveals that ETM fits significantly better than Rach Testlet model (critical χ^2^ value is 9.49 for 4 degrees of freedom on *p* < 0.05 significance level; empirical χ^2^ value is 331.39).

The results from Rasch Testlet Model are presented in Table 2. The results indicate that the sample appears to be rather homogeneous in terms of the ability distribution. Relatively small variances of the latent abilities can explain the low reliability of estimates. Variance of specific factors from this model measures a degree of local dependence (Wang and Wilson, 2005). Therefore, it is notable that analogy and antithesis subscales possess more specific variance (LID) than the entire general factor.

**TABLE 2:** Internal structure of country-specific relational reasoning construct from orthogonal Rasch Testlet model.

**Scale**	**Variance**	**EAP reliability**
General RR	0.63	0.60
Analogy	0.75	0.39
Anomaly	0.47	0.22
Antinomy	0.61	0.32
Antithesis	0.83	0.46

The results from the ETM are presented in Table 3. The results suggest that the variance of three components of RR lowered compared to their estimates from orthogonal Rasch Testlet Model (analogy, anomaly, and antithesis). However, the variance of the fourth component (antinomy) increased. Notably, the variance of the general RR did not change across the models, but its reliability increased. We emphasize that the interpretation of factors differs across these models because of the difference in the modeled structures.

**TABLE 3:** Internal structure of country-specific relational reasoning construct from the ETM.

**Scale**	**Correlation with general RR**	**Variance**	**EAP reliability**
General RR	–	0.63	0.67
Analogy	0.27*	0.34	0.45
Anomaly	−0.09*	0.17	0.12
Antinomy	−0.70*	0.76	0.46
Antithesis	–0.02	0.63	0.38

*p < 0.001.

The results from the ETM suggest that the better engineering students perform in general on RR, the worse they are at defining criteria to distinguish continuums (antinomy scale). However, this exact subscale describes a measure of the ability to identify compromises between different solutions (Dumas and Schmidt, 2015). This may be a sign of potential difficulties in future engineering performance for students. At the same time, positive relations between the overall reasoning and analogical reasoning have been identified in several previous studies (Carpenter et al., 1990) and demonstrated here. However, relations among other forms of RR and general RR itself are negative or insignificant, suggesting that these parts of RR do not relate to it in any way that cannot be explained by other subscales (that is, controlling for other subscales).

The last portion of the results came from the GSM (Table 4). Note that these results are averaged across four recalibrations of the model (skipping every specific factor from calibration). However, the maximum difference between the same parameter across different recalibrations is less than 0.02. The results suggest that this model provides overall the most balanced and reliable estimates of a general RR general as well as its specific factors. That is, although variances of latent variables are not the biggest across the three considered models, the reliability of them appears to be optimal. Notably, the general RR returns the highest reliability under the GSM structure along with shrinking its variance. However, the variance of antinomy subscale reaches its peak in this model, implying that this scale measures cognitive skill distinct from general RR. Patterns of correlations of latent variables support this conclusion.

**TABLE 4:** Internal structure of country-specific relational reasoning construct from the GSM.

**Scale**	**Scales**			**Variance**	**EAP reliability**
	**Analogy**	**Anomaly**	**Antinomy**		
General RR	–	–	–	0.57	0.75
Analogy				0.24	0.39
Anomaly	0.32*			0.25	0.35
Antinomy	−0.55*	-0.70*		0.86	0.44
Antithesis	−0.21*	-0.03	-0.53*	0.36	0.45

*p < 0.001.

These relationships may indicate how students achieve a score on general RR. The abilities to find anomalies and analogies are positively correlated. It is possible to conclude that these abilities share, to some extent, the same cognitive processing: to define which elements are to be excluded, one should define what is similar among other elements. Interestingly, scores on the anomaly subscale do not depend on scores on the antithesis subscale: the ability to define an outlying sign of a breaking pattern does not relate to the ability to find the opposite pattern.

## Discussion

Relational reasoning is believed to be an essential construct for studying higher education learning. Nature of RR reflects the ability of an individual to capture complex relations between patterns within the stream of information. Accordingly, RR can be conceptualized in a multitude of forms, based on the content of information (e.g., professional knowledge or common sense), its type (verbal, numerical, graphical), complexity of relations (e.g., number of analyzed rules), or kind of relations (such as resemblance or divergence). The analyzed TORR conceptualizes it in four types of relations connecting abstract geometric patterns: analogy (similarity), anomaly (discrepancy), antinomy (incompatibility), and antithesis (polarity; Alexander et al., 2016a; Dumas and Alexander, 2016). Many studies proved its predictive power and importance, and the TORR itself has been shown to exhibit good psychometric properties.

However, studying the nature of RR has been limited by the traditions of psychometric modeling. Because RR itself has a composite nature, researchers applied bifactor models to describe it. As a result of this, extracted factor scores do not correlate with each other because of technical necessity. For the case of the TORR, this means that scores on the analogy subscale are not related to general RR; nor are they related to any other subscale. However, analogical reasoning is regarded as the basis of cognitive processing (Gust et al., 2008). Therefore, at least this subscale should be correlated with general RR as well as with other subscales.

Bifactor modeling techniques require severe constraints to be forced on relations of latent variables: they are assumed to be orthogonal. As a result of this, their interpretation becomes sophisticated and barely useful for practitioners (Bonifay et al., 2017). That is, interpretation of specific factors implies that they do not contain any information, described by the general factor; nor do they contain information described by other specific factors. Consequently, the domain of bifactor models usually is limited by the separation of the general factor from contexts of its manifestations. Primordial example of this is modeling LID, caused by shared stimuli of items (DeMars, 2006). Within this example, subscores do not possess any meaningful interpretation from the beginning and are extracted only to reach local independence of items on person parameters. This is, clearly, not the case for composite psychological constructs, where components have meaningful interpretation and cannot be expected to be orthogonal.

Oblique bifactor models can be considered to overcome these limitations. These models allow relaxing the assumption of total orthogonality traditionally required for bifactor modeling. The set of these models includes (but is not limited to) (1) the ETM (Paek et al., 2009) and (2) the GSM (Brandt, 2017). While the ETM allows specific factors to correlate with the general factor but not with each other, the GSM allows them to correlate with each other but not with the general factor. As a result of this, these models extract general factors that differ in interpretation and psychological meaning but allow researchers to study the inner structure of psychological constructs. However, these models do not exhaust the set of oblique bifactor models; e.g., one can conceive models with zero constraints on the sum of some or all values in the variance–covariance matrix (e.g., Robitzsch et al., 2020). Nonetheless, the interpretation of such models is next to impossible because it is next to impossible to have theoretical expectations of this kind. It appears such models can only be used to improve model fit in the case when the orthogonal bifactor model exhibits inappropriate model fit. Despite that, further investigation of oblique bifactor models appears to be promising. Such further research include other constraints on the variance–covariance matrix (including nonzero constraints on the sum of its values) and using strong priors about variance–covariance values in the Bayesian paradigm.

For the TORR example, the GSM is the best-fitting model. This means that after extraction of the general RR subscales preserve some relations between each other. Also, these correlations are more important than correlations of the subscales with the general RR. This means that the manifestations of the RR differ more significantly in their relation to each other, whereas their relation to the RR is more homogeneous. Moreover, the assumption of their orthogonality leads to misspecification of the measurement model. Combining results of the ETM and the GSM, several conclusions arise. First, cognitive processing of analogies is the basis of RR, as well as other intellectual activities (Carpenter et al., 1990; Gust et al., 2008). Second, students of engineering programs can increase their total RR scores by having higher scores of one of analogy and anomaly, antinomy, or antithesis abilities. Because this indicates, to some extent, mutually exclusive groups of cognitive abilities, a possible investigation of these results may be directed profiling of cognitive abilities. Third, the most outlying manifestation of RR is antinomy. It correlates negatively to negligibly with other components of RR and the general RR itself. More in-depth investigation of this cognitive process is of great interest.

Unfortunately, the TORR subscores from oblique bifactor models appear to be unreliable, as well as from orthogonal bifactor model. Although this may not be the case for other instruments, this is a natural result for bifactor modeling (Haberman and Sinharay, 2010). However, for some purposes, it is required to have specific subscores with reliable estimates. There are several ways to do so. One of them is recalibrating data within correlated factors model and defactor ignoring model fit indices. This approach is unpopular in the statistical literature, although it fits to willingness to not restrict interpretation to a single model (Organization for Economic Co-operation and Development, 2005; Brandt et al., 2014). Another approach is the application of the composite model, which combines reflective and formative approaches within a single model (Wilson and Gochyyev, 2020). However, this model is more or less equivalent to the correlated factors model and therefore describes the same relations between subscales. While bifactor models *extract* the general factor from the subscales, the composite model *distributes* it across them in the same manner as models without general factor do. As a result of this, it provides high estimates of reliability for subscores.

Several significant limitations cannot be ignored. In this study, we did not discuss the TORR comparability across various demographics groups, for two reasons. The first reason is regarding the graphical nature of the test and therefore the plausible assumption for item comparability. Second, previous studies revealed decent item-level cross-demographics comparability of the TORR in terms of race and gender (Dumas, 2016; Dumas and Alexander, 2018). However, those demographic groups were sampled inside the United States. Therefore, cross-national comparability of the TORR remains unknown. Nevertheless, studying cross-national comparability in terms of item behavior is possible using modifications of the orthogonal bifactor model that allow for the decomposition of differential item functioning into testlet-based and item-based components (Paek and Fukuhara, 2015; Fukuhara and Paek, 2016). Applications of this approach to enhanced bifactor models and changes in their interpretation are of interest. Nonetheless, since the topic of comparability lies beyond the scope of this article, we did not test it. Another limitation concerns the interpretation of subscores and their relations. Although they can be described in terms of original names of the subscales, further theoretical and, probably, experimental study of subscales purified from general RR and subscales purified from each other is required. We also did not consider higher-order model. Even this model is nested within the same class of hierarchical models as bifactor models (Yung et al., 1999; Rijmen, 2010), they reflect latent structures, which can be analytically inferred from the correlated factors model without general factor. Therefore, the second-order models are vulnerable to the positive manifold effect. Moreover, they do not imply the use of specific factor scores, which makes them less useful for practitioners.

Probably, the most significant limitation of this study concerns the application of only Rasch-type models. The used oblique bifactor models were proposed and studied only within Rasch modeling approach. This guarantees that these models return unbiased estimates. Moreover, Rasch modeling setup provides numerical stability, which is desirable for such heavily parametrized models as oblique bifactor models. However, the counterparts of the described models can be conceived within 2PL (Birnbaum, 1968) and, probably, other IRT models. Rasch modeling imposes strict assumptions regarding item discrimination parameters. On the one hand, it guarantees that the probability of solving an easier item is always (on any level of ability) higher than the probability of solving a harder item. This allows for a straightforward interpretation of parameters and facilitates the development of the continuum of observed behavior. On the other hand, it implicates that all items share an equal portion of variance with corresponding latent variable. This assumption may not be as feasible for psychological constructs as it is for educational constructs. Therefore, replication of this study under IRT models with more parameters per item is of interest. Given, of course, that oblique bifactor models are as well-behaved under those IRT models as under Rasch modeling framework.

## Data Availability Statement

The raw data supporting the conclusions of this article will be made available by the authors, without undue reservation.

## Ethics Statement

Ethical review and approval was not required for the study on human participants in accordance with the local legislation and institutional requirements. The patients/participants provided their written informed consent to participate in this study.

## Author Contributions

DF conducted the analyses and wrote the manuscript.

## Conflict of Interest

The authors declare that the research was conducted in the absence of any commercial or financial relationships that could be construed as a potential conflict of interest.
